# Single-Centre Analysis of Magnetic Resonance Imaging of Sacroiliac Joints in a Paediatric Population

**DOI:** 10.3390/jcm13237147

**Published:** 2024-11-26

**Authors:** Joanna Ożga, Monika Ostrogórska, Wadim Wojciechowski, Zbigniew Żuber

**Affiliations:** 1 Department of Pediatrics, Faculty of Medicine, Andrzej Frycz Modrzewski Krakow University, Gustawa Herlinga-Grudzińskiego 1, 30-705 Krakow, Poland; 2 Clinical Department of Pediatrics and Rheumatology, St. Louis Regional Specialised Children’s Hospital, Strzelecka 2, 31-503 Krakow, Poland; 3 Department of Radiology, Jagiellonian University Medical College, Botaniczna 3, 31-503 Krakow, Poland

**Keywords:** sacroiliac joints, MRI, sacroiliitis, juvenile idiopathic arthritis, bone marrow edema

## Abstract

**Background:** Sacroiliitis in children is usually connected with one of the subtypes of juvenile idiopathic arthritis (JIA), such as enthesitis-related arthritis, psoriatic arthritis, or undifferentiated arthritis. The main diagnostic method is magnetic resonance imaging (MRI) of the sacroiliac joints, which can reveal bone marrow edema (BME) as a sign of an active inflammation process. This research aimed to retrospectively investigate the associations between the clinical presentation, laboratory test results, and MRI results of the sacroiliac joints of children. **Methods:** A total of 152 paediatric patients who underwent MRI of the sacroiliac joints were included in this single-centre study. The mean age of patients was 13.91 ± 2.97, while the female-to-male ratio was 1.36:1. JIA diagnosis was confirmed in 91 (59.87%) patients. **Results:** The main symptom reported by 128 (83.21%) patients was chronic pain, while in another 40 (31.25%) patients, it was chronic back pain. Patients with arthritis and BME in the sacroiliac joints were more likely to report chronic back pain, while patients with JIA but without BME in the sacroiliac joints were often positive for anti-nuclear antibodies (ANA). The widening of any joint contour was observed in 43 (28.29%) patients, and reduced joint mobility was observed in 61 (40.13%). Elevation of inflammatory blood parameters occurred in 31 (20.39%) patients, but this was not statistically related to BME presence in the sacroiliac joints. Radiological findings included BME (n = 36; 23.68% of examinations), joint space narrowing (n = 10; 27.78% of examinations), erosions (n = 7; 19.44% of examinations), and joint fluid (n = 7; 19.44% of examinations). There was a statistically significant relationship between the presence of BME in the sacroiliac joints and all of the above radiological findings. **Conclusions:** The radiological findings of MRI of the sacroiliac joints are significantly statistically related to chronic back pain in patients, while there is no relationship between any inflammatory blood parameter and the presence of BME.

## 1. Introduction

Juvenile idiopathic arthritis (JIA) is the most common rheumatic disease in children, with a prevalence of approximately one in a thousand and an incidence between two and twenty-eight per hundred thousand children [1]. JIA is defined by the International League of Associations for Rheumatology (ILAR) as chronic arthritis of unknown origin lasting for at least six weeks and beginning before the age of 16 years [2]. The main symptoms of JIA include chronic pain, limitation of motion and swelling of the joints, mobility difficulties, recurrent fever, fatigue, weight loss, and growth failure [3]. There are seven mutually exclusive subtypes of JIA, defined based on clinical presentation and laboratory and radiological test results. Sacroiliitis encompasses three subtypes of JIA: enthesitis-related arthritis (ERA), psoriatic arthritis (PsA), and undifferentiated arthritis. These three JIA subtypes may alternatively be referred to as juvenile spondyloarthropathies (JSpA). Children with JSpA have varying degrees of enthesitis symptoms and arthritis with axial joint involvement [2].

Sacroiliitis is an inflammation of the sacroiliac joints, and its first symptom is usually a specific pain localised in the lower back that can extend down the legs [4] when patients are sitting down, walking for long periods of time, climbing stairs, or laying on the affected side [5,6]. However, this chronic back pain is not often present in children [7,8,9]. For this reason, to properly assess a patient’s condition, it is essential to perform imaging procedures such as magnetic resonance imaging (MRI), computed tomography (CT), or X-ray [10]. MRI of the sacroiliac joints is the most sensitive method for detecting the early onset of sacroiliitis [11] and consequently administering the proper treatment before the occurrence of irreversible joint destruction.

The aim of this study was to evaluate whether MRI of the sacroiliac joints is able to facilitate the diagnosis of rheumatic disease in paediatric patients and to determine whether these findings correlate with blood inflammatory markers such as elevated leucocyte count, C-reactive protein (CRP), erythrocyte sedimentation rate (ESR), human leucocyte antigen (HLA) class I molecule B27 and Cw6, anti-nuclear antibodies (ANA), and rheumatoid factor (RF).

## 2. Materials and Methods

### 2.1. Patients

In this single-centre retrospective study, 152 paediatric patients hospitalised between November 2021 and June 2024 in the Department of Paediatric Rheumatology in Kraków, Poland, who underwent MRI of the sacroiliac joints were included. The main inclusion criterion was the performance of MRI of the sacroiliac joints. The criteria for MRI assessment was the presence of chronic back pain but also a positive family history of arthritis or psoriasis and the presence of uveitis. The analysis was based on previously anonymized data from the hospital’s database and consisted of MRI of the sacroiliac joints, clinical observations, laboratory tests results, and final diagnosis. The research was conducted in accordance with the Declaration of Helsinki and obtained approval from the Institutional Bioethics Committee (No. of approval: KBKA/52/O/2023, date of approval: 21 September 2023).

### 2.2. Scale for Assessment of Joint Involvement

To assess joint involvement in patients, the researchers introduced a standardised scale. The scale for assessment of joint involvement considers the presence of pain, swelling, and limitation of motion (LOM) in each joint. The patient receive one point for the presence of each of these features. However, there are the following exceptions: in the peripheral joints, only pain and swelling were assessed in the sternoclavicular joints; a common point for LOM was given for the acromioclavicular and shoulder joints; and in the hip joints, points were only given for pain and LOM. Additionally, regarding axial involvement, one point was given for pain in each of the sacroiliac joints, while in the cervical, thoracic, and lumbar spine, one point was given for pain and one for LOM without differentiating between the sides. The minimum score that can be obtained on this scale is zero points while the maximum is two hundred and twelve points. The scale for the assessment of joint involvement also distinguishes between the side on which the joints are involved and whether it is axial or peripheral involvement. An image depicting the whole scale for the assessment of joint involvement is provided in Supplemental Materials. Based on this, a shortened version of the table depicting the scale for the assessment of joint involvement summarised for all patients was produced and used to present the final results.

### 2.3. Laboratory Tests

The laboratory tests performed on patients included CBC and assessments of inflammatory parameters such as ESR and CRP. Each patient also underwent tests assessing liver and kidney function such as AST, ALT, GGTP, ALP, and creatinine, as well as those assessing muscle function, such as LDH and CK. Due to the specific profile of patients, tests for rheumatic diseases were also conducted, including those testing for ANA, RF, HLA-B27, and HLA-Cw6 presence.

### 2.4. MRI Protocol and Interpretation of MRI Findings

**Figure 1:**
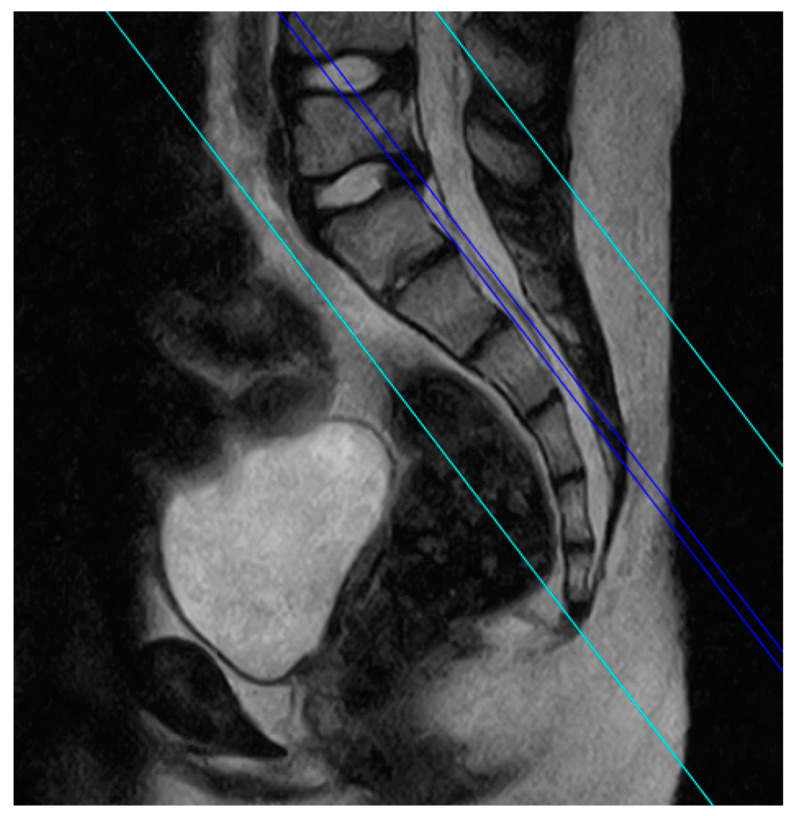
MRI examination of the sacroiliac joints of a 10-year-old male patient conducted using a 0.4 Tesla scanner in T2-weighted sequence presenting the appropriate position to acquire the relevant slices of the examined joints.

All patients underwent initial sacroiliac MRI with contrast media administration due to suspected rheumatic disease. Examinations were performed on both a 0.4 Tesla MRI scanner (Fujifilm, APERTO Lucent) and a 3.0 Tesla MRI scanner (Achieva, Philips Healthcare). Of the examinations performed, 102 (67.11%) were conducted with the 0.4 Tesla MRI scanner, while the remaining 50 (32.89%) examinations were conducted with the 3.0 Tesla MRI scanner. The institutional protocol developed for the assessment of MRI of the sacroiliac joints in the paediatric population was used. In lower-field-intensity MRI, an optimized protocol was used to adjust scanning parameters in order to obtain the highest possible image quality. The patients remained in one position with free breathing during the whole procedure. The scanners were adjusted to obtain the scans in the sagittal plane and in the oblique coronal plane, determined to be a plane parallel to the line drawn between the posterior edges of the S1 and S2 vertebrae (Figure 1). MRI examinations were acquired in T1FFE (T1-weighted gradient echo), T2TSE (T2-weighted ultra-fast spin echo), and STIR (short tau inversion recovery, also known as T2-weighted sensitive to water) sequences.

All examinations were analysed by a radiologist with over 20 years of expertise in musculoskeletal MRI. The review focused on identifying bone marrow edema (BME), joint space narrowing, and the presence of synovial fluid or erosions.

BME was defined, in accordance with the ASAS classification criteria, via the presence of visible-on-STIR-sequence hyperintense areas in the sacral interforaminal bone marrow (brighter areas). Sacroiliitis can be diagnosed if more than one BME lesion is present on a single MRI slice or when it is present on at least two consecutive MRI slices. The lesions must be clearly visible and located in a typical anatomical area, i.e., subchondral bone [12,13,14,15,16].

## 3. Statistical Analysis

All data were analysed using Statistica 13.3 (StatSoft). All data were considered for patient groups regarding JIA diagnosis and the presence of sacroiliitis on the MRI scans. Descriptive statistics methods were used for all obtained laboratory test results and MRI results. Qualitative statistics included chi-squared tests (χ^2^ tests) for comparison according to sex, the presence of chronic back pain, HLA-B27/HLA-Cw6/RF/ANA results, the presence of erosion or joint space narrowing, and the presence of arthritis. The correlation of leucocyte, ESR, and CRP levels with age was verified by linear regression analysis. The Mann–Whitney U test was used for comparison according to age and leucocytes/ER/CRP results in patients regarding the presence of BME. The presence of both arthritis and BME and patient age were examined with ANOVA. Post-hoc Bonferroni correction was used if the ANOVA results were statistically significant. The statistical significance for each analysis was defined as *p* < 0.05.

## 4. Results

### 4.1. Demographic Data

The studied population consisted of 88 (57.89%) females and 64 (42.11%) males. The mean age of patients was 13.91 ± 2.97, with a minimum value of 5 and a maximum of 17 years. The majority of patients (91, 59.87%) fulfilled the ILAR criteria [2] for the diagnosis of JIA, of which 54 (59.34%) were female. The division into JIA subtypes was as follows: thirty-six (39.56%) patients were diagnosed with ERA, thirty (32.97%) with oligoarticular JIA, eighteen (19.78%) with seronegative polyarticular JIA, six (6.59%) with juvenile psoriatic arthritis, and one (1.1%) with systemic-onset JIA. The population was divided into three groups taking into consideration JIA diagnosis and the presence of sacroiliitis on the MRI scans. The first group included patients with JIA and BME presence in the sacroiliac joints, while the second included patients with JIA and an absence of BME in the sacroiliac joints, and the third included patients without JIA or BME in the sacroiliac joints. Group 3 consisted of patients who did not meet the ILAR criteria [2] for the diagnosis of JIA after laboratory tests and MRI of the sacroiliac joints. Some of the patients in this group, due to the presence of joint pain, swelling, and/or reduced joint mobility, were discharged from hospital with recommendations to remain under the supervision of the rheumatology and orthopaedic clinics. There were no patients without arthritis with the presence of BME in the sacroiliac joints. Group 1 consisted of 36 (23.68%) patients, Group 2 of 55 patients (36.18%), and Group 3 of 61 patients (40.13%). The mean age in each group was as follows: 13.69 ± 3.26 (Group 1); 14.22 ± 2.85 (Group 2); 13.77 ± 2.92 (Group 3). There were no statistically significant differences in patient age nor sex distribution between the groups.

### 4.2. Clinical Presentation

The symptom reported by patients most frequently in 128 (84.21%) cases was chronic pain in any of the joints, while chronic back pain was reported by 40 (31.25%) of these patients. Chronic pain was defined as pain lasting longer than 12 weeks that has not discontinued, even after the healing of the initial injury or underlying cause of the acute pain. Chronic back pain was reported by 17 (47.22%) patients in Group 1, 10 (18.18%) patients in Group 2, and 13 (21.37%) patients in Group 3. Patients with arthritis and the presence of BME in the sacroiliac joints (Group 1) were statistically significantly more likely to report chronic back pain than other studied patients. Pain was more frequently localised in the lower limbs (101 cases) than in the upper limbs (35 cases). Physical examination revealed the widening of any joint contour in 43 (28.29%) patients, which was localised in the lower limbs of 28 patients and in the upper limbs of 19 patients. Reduced joint mobility was observed in 61 (40.13%) patients, and in 48 cases, this involved the joints of the lower limbs, while in 14 cases, it involved the joints of the upper limbs. Detailed results of the joint assessment using the standardised scale for the assessment of joint involvement are presented in Table 1, Table 2, Table 3 and Table 4. The tables present the number of patients with involvement (i.e., pain, swelling, or limitation of motion (LOM)) of at least one of the peripheral joints (with a distinction between the right and left side) or the axial joints, along with information on the total number of patients with at least one of the joints impaired in the last row. A positive family history of psoriasis was reported in 12 (7.89%) patients.

### 4.3. Laboratory Findings

**Scheme 1:**
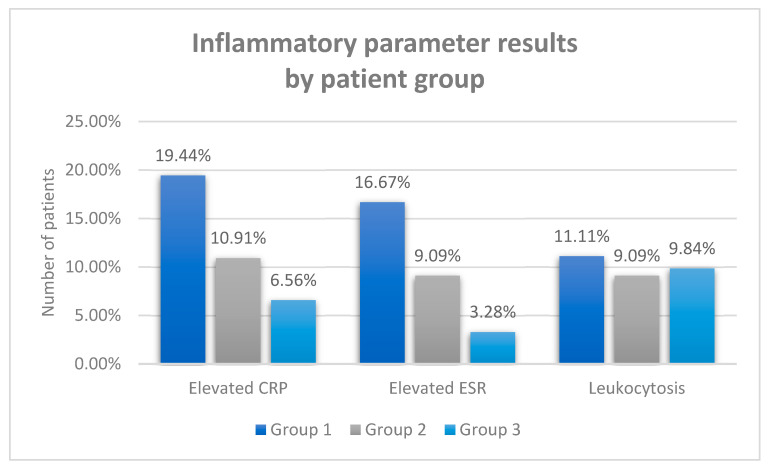
Distribution of the laboratory findings regarding inflammatory parameters by patient group.

Laboratory tests results revealed an elevation of inflammatory parameters, such as CRP, ESR, or leucocytes, in thirty-one (20.39%) patients (Group 1: nine patients (25%); Group 2: eleven patients (20%); Group 3: eleven patients (18.03%)). Increased CRP was found in 17 (11.18%) patients and increased ESR in 13 (8.55%) patients, while leucocytosis was present in 15 (9.87%) patients. There was no statistically significant correlation between patients’ age and inflammatory parameters levels in any of the groups. Thrombocytosis was noted in four (2.63%) patients. Elevation in markers such as ALT occurred in 31 (20.39%) patients: high AST was found in 36 (23.68%) patients and high GGTP in 13 (8.55%) patients. Creatinine was increased in 18 patients (11.84%). ALP was decreased in four (2.63%) patients and increased in seventy-one (46.71%) patients. LDH levels were elevated in five (3.29%) patients, while CK was within the reference values across the whole studied population. Detailed results of the laboratory findings regarding inflammatory parameters are presented in the Scheme 1. There was no statistically significant difference in leucocytosis or ESR or CRP levels between the studied groups.

The results of specific tests performed due to rheumatic disease suspicion are presented in Table 5. In addition, RF levels were increased only in one (0.66%) patient who had not met diagnostic criteria of JIA. In Group 2, there were statistically significantly more patients with positive ANA than in the remaining two groups, while there were no such relationships for the positive results of RF, HLA-B27, or HLA-Cw6.

### 4.4. MRI Findings

**Scheme 2:**
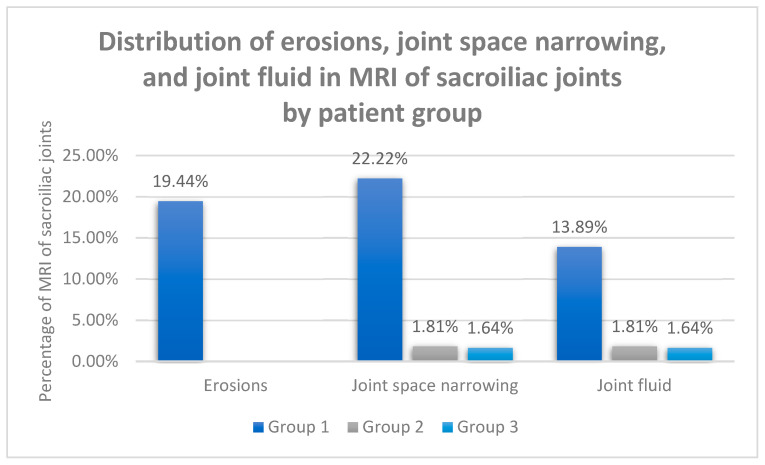
Distribution of erosions, joint space narrowing, and joint fluid in MRI of sacroiliac joints by patient group.

In the MRI of the sacroiliac joints of thirty-six (23.68%) patients, BME was found; in seven (4.61%) cases, the MRI depicted a previous presence of sacroiliitis; meanwhile, eighty-eight (57.89%) examinations proved to be normal. BME was found in 17 patients (42.5%) with chronic back pain history. In 23 (63.89%) cases, the BME was localised bilaterally. It was more frequent in the right sacroiliac joint (31:28 cases). Diffuse BME was described in 12 (33.33%) cases, while discrete BME occurred in 24 (66.67%) cases. In addition, joint space narrowing was found in ten (27.78%) patients, and the presence of erosions or joint fluid were each found in seven (19.44%) cases. The distribution of presence of erosions, joint space narrowing, and joint fluid is shown on Scheme 2.

**Figure 2:**
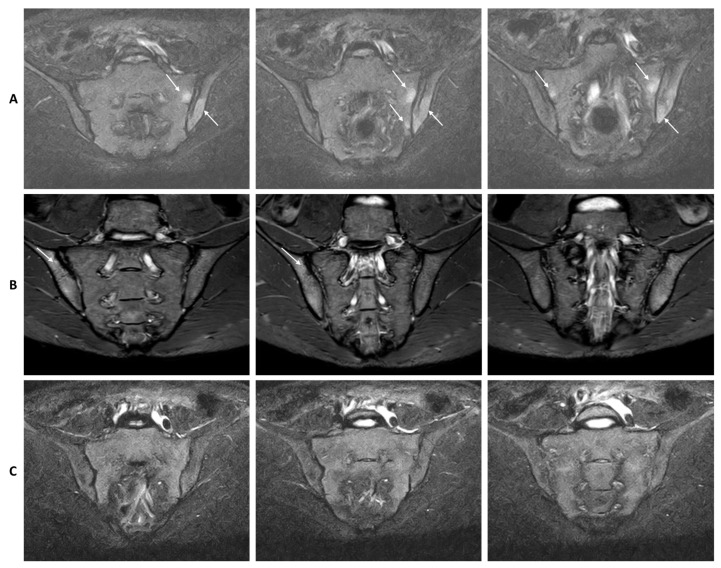
(**A**). MRI of the sacroiliac joints of a 17-year-old female patient performed using a 0.4 Tesla scanner in STIR sequence. On three consecutive slices, diffuse areas of BME can be noted bilaterally in the lateral sacrum and on the left side in the iliac bone, indicated by arrows. (**B**). MRI of the sacroiliac joints of a 17-year-old male patient performed using a 3.0 Tesla scanner in STIR sequence. On the first two consecutive slices, subchondral areas of sclerotisation with marginal BME can be noted on the right iliac bone, indicated by arrows. In addition, small erosions appear in both joints at the level of the visible sclerotisation. (**C**). Normal MRI of the sacroiliac joints of a 17-year-old female patient performed using a 3.0 Tesla scanner in STIR sequence.

The presence of BME in the sacroiliac joints was significantly statistically more often visible in patients with erosions, joint space narrowing, and joint effusion in the same location. Figure 2 presents three MRI scans of sacroiliac joints with BME.

## 5. Discussion

MRI of the sacroiliac joints of children is an imaging method widely used in the diagnosis of sacroiliitis due to its ability to detect BME [17,18]. It provides the possibility of diagnosing sacroiliac joint involvement at an early stage and can thus provide the opportunity for the implementation of pharmacological treatment to prevent irreversible joint damage. This is particularly important in the paediatric population, as failure to introduce effective treatment promptly can result in disability and affect quality of life in the future. In the studied population, 31.25% of patients reported chronic back pain, while BME in the sacroiliac joints was found in 42.5% of these patients. Moreover, patients in Group 1 were more likely to have a history of chronic back pain, which indicates that all patients who report this kind of pain should undergo MRI of the sacroiliac joints.

It should be noted that because of the study’s limitation, which is its retrospective nature, a scale for the clinical assessment of the JIA patients’ condition such as cJADAS (clinical Juvenile Idiopathic Arthritis Disease Activity Score) could not have been applied [19]. For this reason, the scale for the assessment of joint involvement created by the authors and based on the available medical data was used. Furthermore, as the researchers aimed to standardize the clinical assessment of the patients in order to provide a comparison between the groups, the scale was based on the evaluation of pain, swelling, and LOM. The collected results allowed the peripheral and axial joint involvement to be shown in Table 1, Table 2, Table 3 and Table 4.

The involvement of the sacroiliac joints in the course of JIA allows the diagnosis to be directed towards variations of JSpA such as ERA, PsA, or, in particular cases, the undifferentiated arthritis referred to as ILAR [2]. The detection of active inflammation via MRI of the sacroiliac joints allows the selection of appropriate treatment [20] and modifies the prognosis [21] and classification of the disease in adulthood.

In the paediatric population, the assessment of MRI scans of the sacroiliac joints can be very challenging due to growth-related changes that can mimic BME [17,18]. In adults, research studies have been conducted on the anatomical distribution of sacroiliac joint lesions, which might be potentially applicable to children as well, in order to distinguish among other similar lesions that do not meet BME criteria [22]. For this reason, not only should the assessment of paediatric MRIs be performed by a radiologist with extensive experience, but it should be acknowledge that it is more difficult to create fully automated algorithms for BME detection in children. One limitation of this study is that both a high-field scanner with a field strength of 3.0 Tesla and a low-field scanner with a field strength of 0.4 Tesla were used to perform MRI of the sacroiliac joints. This was due to the availability of the scanners, and as the study is retrospective and the researchers aimed to investigate as many MRIs as possible, it was decided to include all of them. Low field strength can result in reduced image quality, but, as in this case, with current technology and appropriate calibration, it is possible to obtain adequate images to assess even small BME. In addition, there are further advantages of the 0.4 Tesla field strength, such as the increased safety of the examination, its shorter time and therefore reduced risk of motion artefacts, and the decreased necessity to sedate patients during the whole procedure. Artificial intelligence (AI) could facilitate and reduce the time needed for MRI evaluation, but for now, existing algorithms are used only for the assessment of sacroiliac joints in adults [23,24,25]. This is also connected to the fact that AI needs a comprehensive database in order to be well-trained and not give false-positive results, which could result in the unnecessary treatment of patients. The extensive database of paediatric MRI of sacroiliac joints could be collected into a multi-centre survey or data could be collected over a longer period of time. A limitation of this study is its single-centre design, which results in a limited group of patients being included in the study. Subsequently, this group is further divided into smaller subgroups in order to be compared. In order to train the already developed algorithms correctly, more examinations with the presence of sacroiliitis are required. However, it should be noted that only 36 such examinations were obtained in our institution over a period of 32 months.

In our study, the presence of BME in the sacroiliac joints was not found to be related to leucocytosis nor elevations in ESR or CRP, which is not unusual as inflammatory blood markers can be normal in patients with JIA. A possible explanation for the low percentage of children with increased inflammatory blood markers in Group 1 and Group 2 is that they often receive NSAIDs (nonsteroidal anti-inflammatory drugs) prior to hospitalisation in the paediatric rheumatology department. Another reason is that all MRIs of the sacroiliac joints performed at our clinic were included in the study, and consequently, some of the patients had already received treatment for JIA. In contrast, similar studies conducted on adult patients have shown correlations between the findings of MRI of sacroiliac joint involvement and CRP values [26], ESR values, or the sum of CRP and ESR [27]. It is worth noting that in this paper, the population of patients consisted of 152 children, while in studies by Bradella or by Jee, the number of patients was below 20. The fact that ANA-positive results occur often in patients with JIA but without BME presence on MRI scans of the sacroiliac joints may be of clinical relevance, as there are papers reporting that ANA-positive patients with JIA may represent a separate subgroup with homogeneous characteristics [28].

## 6. Conclusions

In conclusion, MRI of the sacroiliac joints enables the early diagnosis of sacroiliitis even before the appearance of the first clinical symptoms. Early identification of sacroiliitis is crucial as it leads to timely treatment and thus better management of the inflammatory process, potentially preventing further joint damage and improving long-term outcomes for patients. The clinical symptom connected with BME presence in the sacroiliac joints is chronic back pain. This symptom should serve as an indication that the patient should be referred for an MRI scan of sacroiliac joints. MRI findings suggesting active inflammation in the sacroiliac joints of children show no relationship to inflammatory blood parameters or positive results for RF, HLA-B27, HLA-Cw6, or ANA. However, a positive ANA result is statistically significantly more often visible in patients with JIA but without sacroiliitis. This suggests that MRI is a sensitive tool in detecting early sacroiliac joint involvement in children.

## Figures and Tables

**Table 1:** Detailed results of the joint assessment with the use of the standardized scale concerning the entire study population. Symbol “X” was placed when no points could be given.

All Patients n = 152						
Joints	Number of Patients with Affected Joints					
	Pain		Swelling		Lom	
Peripheral	right	left	right	left	right	left
	94 (61.84%)	86 (56.58%)	38 (25.00%)	22 (14.47%)	47 (30.92%)	42 (27.63%)
	108 (71.05%)		43 (28.29%)		54 (35.53%)	
Axial	66 (43.42%)		X		20 (13.16%)	
Total	125 (82.24%)		43 (28.29%)		61 (40.13%)	

**Table 2:** Detailed results of the joint assessment with the use of the standardized scale concerning patients in Group 1. Symbol “X” was placed when no points could be given.

Group 1 n = 36 Patients with JIA Diagnosis and the Presence of BME in the Sacroiliac Joints						
Joints	Number of Patients with Affected Joints					
	Pain		Swelling		Lom	
Peripheral	right	left	right	left	right	left
	22 (61.11%)	23 (63.89%)	10 (27.78%)	8 (22.22%)	10 (27.78%)	10 (27.78%)
	25 (69.44%)		11 (30.56%)		12 (33.33%)	
Axial	20 (55.56%)		X		7 (19.44%)	
Total	32 (88.89%)		11 (30.56%)		15 (41.67%)	

**Table 3:** Detailed results of the joint assessment with the use of the standardized scale concerning patients in Group 2. Symbol “X” was placed when no points could be given.

Group 2 n = 55 Patients with JIA and the Absence of BME in the Sacroiliac Joints						
Joints	Number of Patients with Affected Joints					
	Pain		Swelling		Lom	
Peripheral	right	left	right	left	right	left
	39 (70.91%)	28 (50.91%)	23 (41.82%)	12 (21.82%)	22 (40.00%)	15 (27.27%)
	44 (80.00%)		26 (47.27%)		23 (41.82%)	
Axial	18 (32.73%)		X		7 (12.73%)	
Total	48 (87.27%)		26 (47.27%)		25 (45.45%)	

**Table 4:** Detailed results of the joint assessment with the use of the standardized scale concerning patients in Group 3. Symbol “X” was placed when no points could be given.

Group 3 n = 61 Patients Without JIA or BME in the Sacroiliac Joints						
Joints	Number of Patients with Affected Joints					
	Pain		Swelling		Lom	
Peripheral	right	left	right	left	right	left
	33 (54.10%)	35 (57.38%)	5 (8.20%)	2 (3.28%)	15 (24.59%)	17 (27.87%)
	39 (63.93%)		6 (9.84%)		19 (31.15%)	
Axial	28 (45.90%)		X		6 (9.84%)	
Total	45 (73.77%)		6 (9.84%)		21 (34.43%)	

**Table 5:** Number of positive ANA, HLA-B27, and HLA-Cw6 results in patients. Statistical significance between groups was determined at * *p* < 0.05 using chi-squared test.

Laboratory Test	Number of Positive Results				
	ALL	Group 1	Group 2	Group 3	*p*-Value
ANA	40 (26.32%)	7 (19.44%)	21 (38.18%)	12 (19.67%)	*p* = 0.021 *
HLA-B27	60 (39.47%)	17 (47.22%)	22 (40%)	21 (34.43%)	*p* = 0.443
HLA-Cw6	29 (19.08%)	5 (13.89%)	11 (20%)	13 (21.31%)	*p* = 0.589

## Data Availability

Data inquiries can be directed to the corresponding author.

## Supplementary Materials

The following supporting information can be downloaded at: https://www.mdpi.com/article/10.3390/jcm13237147/s1. Table S1: The scale for assessment of joint involvement.
