# Supplementary material for: Single-Centre Analysis of Magnetic Resonance Imaging of Sacroiliac Joints in a Paediatric Population
Source: J Clin Med. 2024 Nov 26;13(23):7147. doi: 10.3390/jcm13237147 (PMC11642054; doi:10.3390/jcm13237147)
Supplement: Supplementary file 1 [file jcm-13-07147-s001.zip › jcm-3329384-supplementary.pdf]

Supplemental material

The scale for assessment of joint involvement.

JOINTS	PAIN		SWELL		LOM	
	RIGHT	LEFT	RIGHT	LEFT	RIGHT	LEFT
Temporo-mandibular						
Sterno-clavicular					X	X
Acromion-clavicular						
Shoulder						
Elbow						
Wrist						
MCP I						
MCP II						
MCP III						
MCP IV						
MCP V						
PIP I						
PIP II						
PIP III						
PIP IV						
PIP V						
DIP II						
DIP III						
DIP IV						
DIP V						
Hip			X	X		
Knee						
Ankle						
Subtalar joints						
Intertarsal joints						
MTP I						
MTP II						
MTP III						
MTP IV						
MTP V						
TOE I						
TOE II						
TOE III						
TOE IV						
TOE V						
Sacroiliac joints			X	X	X	X
Cervical spine			X	X		
Thoracic spine			X	X		
Lumbar spine			X	X		

The scale for assessment of joint involvement.
LOM – limitation of motion
MCP – metacarpophalangeal joint
PIP – proximal interphalangeal joint
DIP – distal interphalangeal joint
MTP - metatarsophalangeal joint
